# Classification of 74 facial emoji’s emotional states on the valence-arousal axes

**DOI:** 10.1038/s41598-021-04357-7

**Published:** 2022-01-27

**Authors:** Gaku Kutsuzawa, Hiroyuki Umemura, Koichiro Eto, Yoshiyuki Kobayashi

**Affiliations:** Human Augmentation Research Center, National Institute of Advanced Industrial Science and Technology, Kashiwa, Japan

**Keywords:** Psychology, Human behaviour

## Abstract

Emojis are frequently used by people worldwide as a tool to express one’s emotional states and have recently been considered for assessment in research. However, details regarding the ways in which they correspond to human emotional states remain unidentified. Thus, this study aimed to understand how emojis are classified on the valence and arousal axes and to examine the relationship between the former and human emotional states. In an online survey involving 1082 participants, a nine-point scale was employed to evaluate the valence and arousal levels of 74 facial emojis. Results from the cluster analysis revealed these emojis to be categorized into six different clusters on the two axes of valence and arousal. Further, the one-way analysis of variance indicated that these clusters have six valence and four arousal levels. From the results, each cluster was interpreted as (1) a strong negative sentiment, (2) a moderately negative sentiment, (3) a neutral sentiment with a negative bias, (4) a neutral sentiment with a positive bias, (5) a moderately positive sentiment, and (6) a strong positive sentiment. Therefore, facial emojis were found to comprehensively express the human emotional states.

## Introduction

Emoji, a powerful non-verbal communication tool^1^, is frequently used by individuals globally to express their emotional states as part of daily communication^2^. Recently, it has also been applied to research fields, such as consumer studies, to assess users’ emotional states. For example, a set of human facial emojis has been employed for evaluating food products to understand participants’ preferences^3^. Compared to traditional text-based methods, the use of emojis in such studies has been advantageous, such as with respect to the ease of answering questions and targeting people familiar with different languages. Therefore, a better understanding of how each emoji is associated with respective human emotional states can help accelerate its applicability.

Human emotional states can be plotted on two independent axes: arousal and valence (i.e., the core affect^4^). For example, Jaeger et al.^5^ reported that 33 types of facial emojis (e.g., ) can be classified into three clusters (i.e., positive, neutral, and negative) based on valence. Was and Hamrick^6^ asked participants to rate the values of 105 common Apple emojis using a five-point scale, and found that the median of these values was 3, but that there was some degree of variability across all emojis. However, these studies did not consider both axes. Therefore, how facial emojis are classified on these two axes and how people recognize the arousal and valence levels from emojis remain unclear. It has been reported that the recognition of human expressions is largely based on the eyes, muscular contractions, and other movements (e.g., mouth, eyebrows)^7,8^. However, ordinary emojis without the latest animation features are static anthropomorphic images and cannot represent muscular contractions and other such movements. Therefore, people may refer to different features to recognize the arousal and valence levels from these emojis. Clarifying how emojis are categorized on both the arousal and valence axes using cluster analysis and how people recognize the arousal and valence levels from emojis will allow us to understand the common features of emojis that represent similar emotional states and better interpret the results of studies that use facial emojis to assess emotional states.

Various kinds of facial emojis are considered capable of assessing emotional states. According to Emojipedia^9^, the Apple platform (ver. iOS 14.6) has implemented 94 kinds of human facial emojis thus far. However, in comparison, previous studies have explored an insufficient number of facial emojis. For example, Jaeger et al.^5^ and Phan et al.^10^ used only a set of 33 and 6 facial emotions to assess emotional states, respectively. Therefore, it remains unclear how emojis excluded from the scope of previous studies are categorized on the arousal and valence axes. This clarification would increase the number of emojis considered in research to evaluate human emotional states, thereby facilitating a more detailed analyses.

This study aimed to understand how various facial emojis used by smartphone systems (Unicode 13.0) are classified into the valence and arousal axes. Additionally, it sought to explore the relationship between these emojis and human emotional states based on the core affect theory, and how people recognize arousal and valence levels from emojis. As mentioned above, previous studies have categorized human facial emojis into three clusters on the valence axis. However, they can be plotted on the two independent arousal and valence axes. Thus, we hypothesized that facial emotions could be classified into multiple clusters on these axes.

## Results

### Analyzed data

**Figure 1:**
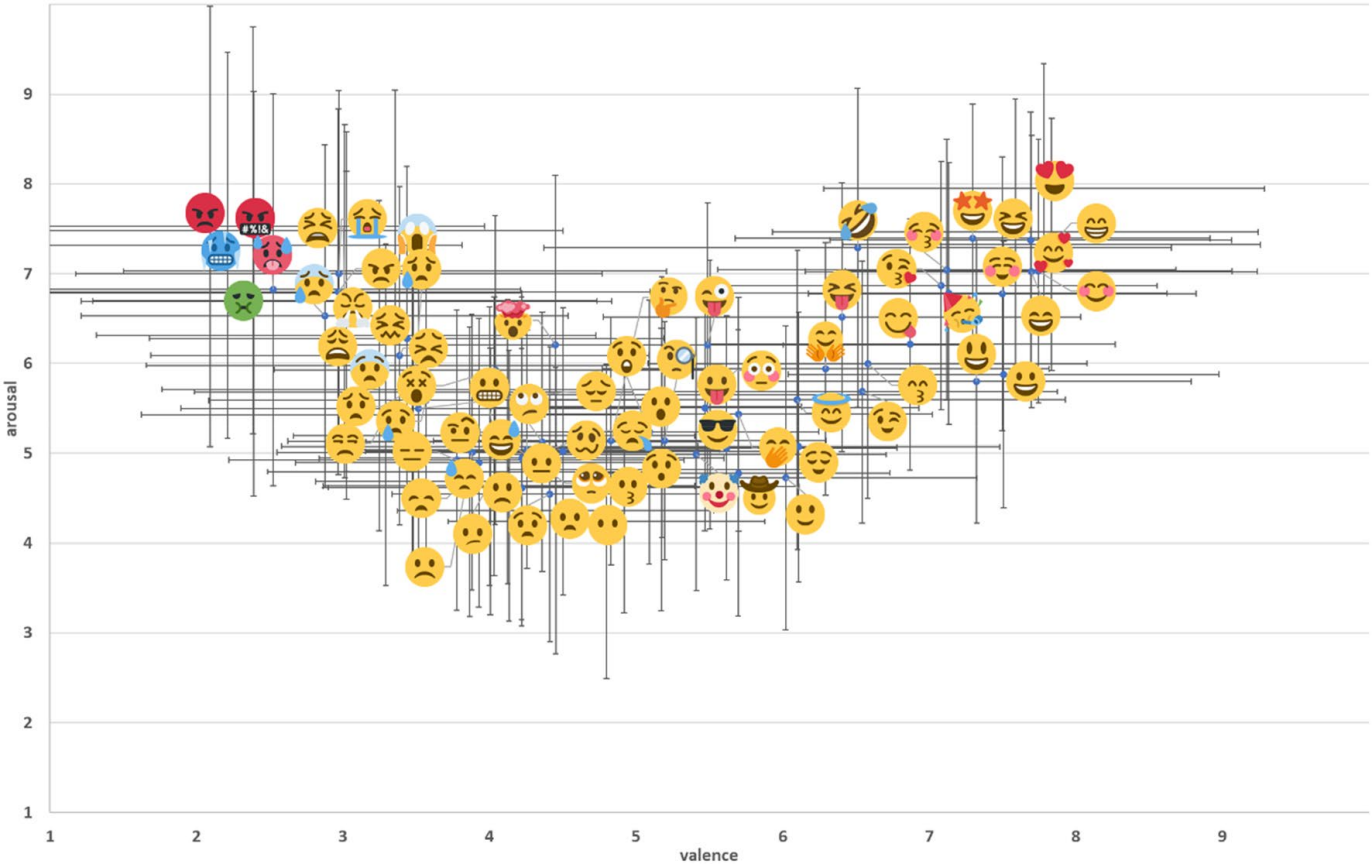
The scatter plot of the mean and standard deviation of the valence and arousal scores for 74 facial emojis evaluated by the participants. The vertical and horizontal axes represent the valence and arousal levels, respectively. The error bars indicate one standard deviation for each variable (i.e., valence and arousal). This figure was created with Microsoft PowerPoint (Microsoft 365 MSO), and the emojis used in the figure are taken from Twemoji (URL: https://twitter.github.io/twemoji/?fbclid=IwAR3bcyVjolPfvFnIvrz4gHTYUDcBlKq8Z_6uQDIGOpiLcZGNejjyEcuBlxU).

The responses to the two dummy questions were examined, and 122 participants who responded incorrectly to both were excluded from the remaining analyses. Consequently, the data of 960 participants could be employed for further analyses (*M* age = 30.55, *SD* = 5.32; 455 males, 505 females). The mean and standard deviation of the valence and arousal levels for each emoji were plotted on a scatter plot (Fig. 1), in which the horizontal and the vertical axes denoted the valence and arousal levels, respectively.

### Clusters of emojis on the valence and arousal axes

A hierarchical cluster analysis was performed on the valence and arousal levels of each emoji to classify similar emojis into several clusters. The Euclidean distance and the Ward aggregation criterion were considered in the analysis (the Z-scores were calculated for each rating and then implemented). The optimum number of clusters was obtained from the dendrogram and the Calinski and Harabasz index^11^, as performed in a previous study^5^. Consequently, a six-cluster solution was retained. Table 1 provides detailed information for each cluster, and Tables 2, 3, 4, 5 display the facial emojis classified into each cluster.

**Table 1:** ANOVA results for mean valence and mean arousal across clusters.

Cluster label	N	Valence	Arousal
Strong positive sentiment	12	7.42 (0.40) a	7.19 (0.34) a
Moderately positive sentiment	9	6.57 (0.62) b	5.98 (0.29) b
Neutral sentiment with a positive bias	12	5.49 (0.38) c	5.19 (0.29) c
Neutral sentiment with a negative bias	19	4.27 (0.38) d	4.83 0.27) d
Moderately negative sentiment	10	3.59 (0.37) e	5.84 (0.30) b
Strong negative sentiment	12	2.74 (0.40) f	6.91 (0.37) a

Post-hoc results (Tukey's test) are shown and clusters with similar letters are not significantly different at 5%. Figures in parentheses represent standard deviations.

**Table 2:** Emoji collection of clusters 1 and 2 (i.e., strong negative sentiment and moderately negative sentiment).

	Name	Emoji	N	Valence	Arousal
Cluster1	Angry face		381	2.97 (2.13)	6.80 (2.04)
	Pouting face		375	2.09 (1.87)	7.53 (2.45)
	Face with symbols on mouth		388	2.39 (2.12)	7.48 (2.27)
	Tired face		382	3.01 (1.72)	6.70 (1.97)
	Weary face		384	3.02 (1.70)	6.31 (1.82)
	Face with steam from nose		379	3.02 (1.81)	6.69 (1.89)
	Face screaming in fear		385	3.36 (1.85)	7.03 (2.02)
	Anxious face with sweat		388	2.88 (1.66)	6.53 (1.91)
	Loudly crying face		379	2.97 (1.80)	7.00 (2.04)
	Nauseated face		384	2.39 (1.83)	6.78 (2.25)
	Hot face		391	2.52 (1.69)	6.82 (2.18)
	Cold face		380	2.21 (1.60)	7.32 (2.15)
Cluster2	Unamused face		383	3.29 (1.67)	5.43 (1.90)
	Worried face		387	3.47 (1.71)	5.71 (1.84)
	Pensive face		385	3.52 (1.62)	5.50 (1.74)
	Persevering face		446	3.39 (1.70)	6.09 (1.89)
	Confounded face		394	3.44 (1.76)	6.28 (1.92)
	Fearful face		381	3.25 (1.59)	5.98 (1.84)
	Crying face		377	3.56 (1.48)	5.58 (1.69)
	Sad but relieved face		388	3.48 (1.50)	5.68 (1.79)
	Dizzy face		383	4.04 (1.51)	5.93 (1.72)
	Exploding head		383	4.45 (1.55)	6.20 (1.89)

The names of the emojis correspond to those registered in Let's emoji. Figures in parentheses represent standard deviations.

**Table 3:** Emoji collection of cluster 3 (i.e., neutral sentiment with a negative bias).

	Name	Emoji	N	Valence	Arousal
Cluster3	Grinning face with sweat		376	4.26 (1.31)	5.04 (1.33)
	Face without mouth		378	4.80 (1.08)	4.24 (1.75)
	Neutral face		391	4.22 (1.32)	4.62 (1.54)
	Expressionless face		384	3.78 (1.55)	4.92 (1.67)
	Face with rolling eyes		389	4.36 (1.20)	5.13 (1.45)
	Face with raised eyebrow		383	4.00 (1.42)	5.08 (1.55)
	Disappointed face		392	3.86 (1.38)	4.79 (1.61)
	Confused face		374	4.01 (1.19)	4.69 (1.49)
	Slightly frowning face		396	4.13 (1.27)	4.64 (1.50)
	Frowning face		390	3.88 (1.33)	5.01 (1.54)
	Grimacing face		379	4.03 (1.37)	5.19 (1.55)
	Pleading face		380	4.12 (1.50)	5.13 (1.58)
	Hushed face		384	5.17 (1.04)	4.82 (1.57)
	Frowning face with open mouth		385	4.45 (1.08)	4.36 (1.59)
	Anguished face		385	4.41 (1.08)	4.55 (1.64)
	Sleepy face		392	4.22 (1.38)	4.94 (1.80)
	Downcast face with sweat		381	3.93 (1.25)	4.90 (1.61)
	Woozy face		386	4.50 (1.35)	5.02 (1.60)
	Kissing face		392	4.92 (1.19)	4.63 (1.41)

The names of the emojis correspond to those registered in Let's emoji. Figures in parentheses represent standard deviations.

**Table 4:** Emoji collection of cluster 4 (i.e., neutral sentiment with a positive bias).

	Name	Emoji	N	Valence	Arousal
Cluster4	Slightly smiling face		386	6.02 (1.31)	4.73 (1.69)
	Relieved face		437	6.11 (1.37)	5.07 (1.50)
	Face with tongue		380	5.47 (1.46)	5.50 (1.36)
	Smiling face with sunglasses		388	5.62 (1.16)	5.06 (1.47)
	Face with monocle		383	5.20 (1.10)	5.14 (1.32)
	Cowboy hat face		396	5.70 (1.04)	4.78 (1.59)
	Clown face		379	5.41 (1.29)	4.99 (1.52)
	Thinking face		385	4.83 (1.21)	5.14 (1.38)
	Face with hand over mouth		372	5.70 (1.32)	5.43 (1.30)
	Flushed face		391	5.50 (1.45)	5.65 (1.50)
	Face with open mouth		392	5.18 (0.95)	5.52 (1.45)
	Astonished face		392	5.09 (1.16)	5.24 (1.47)

The names of the emojis correspond to those registered in Let's emoji. Figures in parentheses represent standard deviations.

**Table 5:** Emoji collection of clusters 5 and 6 (i.e., moderately positive sentiment and strong positive sentiment).

	Name	Emoji	N	Valence	Arousal
Cluster5	Grinning face		388	7.51 (1.47)	5.87 (1.48)
	Grinning face with big eyes		380	7.32 (1.46)	5.80 (1.57)
	Smiling face with halo		375	6.10 (1.83)	5.59 (1.67)
	Winking face		376	6.54 (1.33)	5.68 (1.46)
	Face savoring food		449	6.87 (1.40)	6.21 (1.40)
	Kissing face with smiling eyes		376	6.58 (1.50)	6.00 (1.50)
	Winking face with tongue		458	5.49 (1.82)	6.20 (1.59)
	Squinting face with tongue		387	6.41 (1.63)	6.51 (1.50)
	Hugging face		389	6.29 (1.52)	5.94 (1.41)
Cluster6	Grinning face with smiling eyes		384	7.70 (1.54)	7.02 (1.52)
	Beaming face with smiling eyes		381	7.83 (1.43)	7.32 (1.40)
	Grinning squinting face		383	7.59 (1.66)	7.46 (1.48)
	Rolling on the floor laughing		389	6.51 (2.14)	7.29 (1.78)
	Smiling face		385	7.50 (1.32)	6.77 (1.53)
	Smiling face with smiling eyes		448	7.75 (1.32)	7.03 (1.47)
	Smiling face with heart-eyes		376	7.78 (1.50)	7.95 (1.39)
	Smiling face with hearts		448	7.69 (1.37)	7.37 (1.43)
	Face blowing a kiss		386	7.08 (1.47)	6.87 (1.39)
	Kissing face with closed eyes		383	7.12 (1.56)	7.04 (1.45)
	Partying face		381	7.13 (1.49)	6.78 (1.46)
	Star-struck		377	7.30 (1.62)	7.40 (1.50)

The names of the emojis correspond to those registered in Let's emoji. Figures in parentheses represent standard deviations.

### Characteristics of each cluster in terms of the valence and arousal levels

The one-way analyses of variance (ANOVAs) were performed on the valence and arousal levels to understand the characteristics of each cluster. Among the six clusters, a significant main effect was found on the valence (*F*(5, 68) = 213.88, *p* < 0.001, *η*^2^ = 0.94) and arousal levels (*F*(5, 68) = 126.37, *p* < 0.001, *η*^2^ = 0.90). The Bonferonni correction conducted as a post-hoc analysis demonstrated significant differences among all clusters on the former axis (*p*s < 0.05), thus indicating that they were independent of each other. Furthermore, significant differences (*p*s < 0.05) were found between the clusters: high (clusters 1 and 6), moderate (clusters 2 and 5), low (cluster 4), and very low (cluster 3) (see Table 1 for further details).

## Discussion

This study aimed to assess how various facial emojis are classified in the valence and arousal axes, examine the relationship between these emojis and human emotional states using the core affect theory^4^, and understand how people recognize arousal and valence levels from emojis. Based on the data of 1082 participants, we analyzed the valence and arousal levels indicated by each of the 74 emojis included in our study. These emojis tended to be distributed in a U-shape on the two axes (Fig. 1), which was similar to a previous study’s result that employed only 33 facial emojis^5^. The present study’s emojis were classified into six different clusters on the two axes, as hypothesized initially. Further, these clusters had six valence and four arousal levels (Table 1). Thus, each cluster was interpreted as: (1) a strong negative sentiment, (2) a moderately negative sentiment, (3) a neutral sentiment with a negative bias, (4) a neutral sentiment with a positive bias, (5) a moderately positive sentiment, and (6) a strong positive sentiment. A previous study^5^ categorized emojis into only three levels on the valence axis, possibly because it utilized limited emojis and focused on only one axis constituting the human emotional states. However, the present study confirmed that the emojis could be classified into six clusters on the valence and arousal axes using numerous emojis and that both axes constituted human emotional states. Therefore, it was concluded that emojis could display human emotional states in greater detail than previously reported.

**Figure 2:**
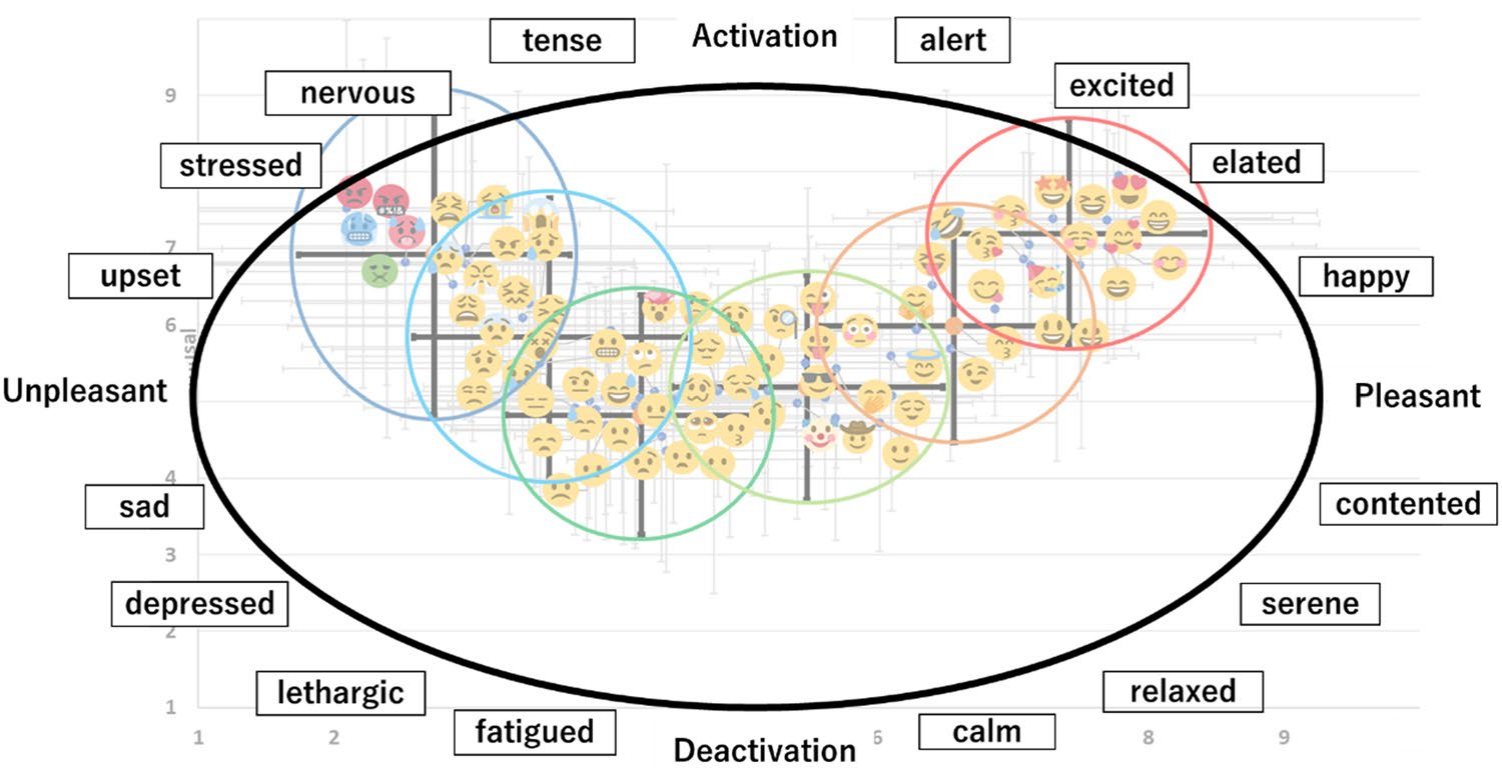
The core affect and each cluster overlaid on the scatter plot of the mean valence and arousal scores for 74 facial emojis. This figure was created with Microsoft PowerPoint (Microsoft 365 MSO), and the emojis used in the figure are taken from Twemoji (URL: https://twitter.github.io/twemoji/?fbclid=IwAR3bcyVjolPfvFnIvrz4gHTYUDcBlKq8Z_6uQDIGOpiLcZGNejjyEcuBlxU).

The human emotional states (the core affect) are circularly aligned on the valence and arousal axes^4^. As this study acquired the valence and arousal levels indicated by each emoji, all six clusters could be corresponded with the emotional states described in the core affect (Fig. 2), which is discussed in the following sections.

In the “strong negative sentiment” cluster, the following 12 emojis were classified: , , , , , , , , , , , and . Many of them contained extra accessories, such as colored (red, blue or green) face, paled expression, or crying face. This cluster was characterized by extremely low valence and high arousal levels (valence: 2.74 ± 0.40, arousal: 6.91 ± 0.37). The human emotional states based on the core affect^4^ that are considered to correspond with such levels would be “nervous,” “stressed,” and “upset.” In present study, emojis named “Confounded face ,” “Tired face ” and “Angry face ” were classified into this cluster. These emojis have also been categorized in the “negative sentiment” cluster, interpreted with the words “nervous/anxious/worried,” “stressed,” and “angry/annoyed,” in a previous study^5^. However, a prior study^5^ included the emoji named “Face screaming in fear ” in the “neutral/dispersed sentiment” cluster. Additionally, it may be interesting to note that this cluster did not have an emoji with the corners of the mouth raised (i.e., smiling). These results indicated that the emojis classified in this cluster corresponded appropriately to the emotional state of the core affect in general; however, the interpretations of some emojis require caution because they are inconsistent across studies.

In the “moderately negative sentiment” cluster, the following 10 emojis were classified: , , , , , , , , , and . Its characteristics comprised low valence and moderate arousal levels (valence: 3.59 ± 0.37, arousal: 5.84 ± 0.30). The human emotional states that were considered to correspond with these levels were “sad” and “depressed.” The present study evidently classified the emojis named “Crying face ” and “Pensive face ” in this cluster. These emojis have also been categorized in the “negative sentiment” cluster, and both interpreted with the words “sad/unhappy” and “depressed,” in a previous study^5^. Furthermore, none of this cluster’s emojis were grouped into the more positive clusters (i.e., “neutral/dispersed sentiment” and “positive sentiment”) in previous studies. Additionally, this cluster did not include the emojis with a raised corner of the mouth (i.e., smiling), similar to those classified into the “strong negative sentiment” cluster. Therefore, it was reasonable to infer that the emojis grouped into this cluster corresponded exceedingly well to the emotional state of the core affect.

In the “neutral sentiment with a negative bias” cluster, the following 19 emojis were included: , , , , , , , , , , , , , , , , , , and . Its characteristics included a low to moderate valence and a very low arousal level (valence: 4.27 ± 0.38, arousal: 4.83 ± 0.27). The human emotional states that were considered to resemble these levels were “lethargic” and “fatigue.” The emojis named “Expressionless face ,” “Neutral face ,” and “Grimacing face ” were grouped into this cluster; they were also categorized in the “neutral/dispersed sentiment” cluster. In a previous research^5^, the former two emojis were interpreted using the words “neutral/indifferent,” “no comments/opinion,” and “confused/unsure” and were considered to be close to the emotional states represented by this cluster. However, the interpretation of the “Grimacing face ” emoji in a previous study^5^ included both positive (i.e. “happy” and “excited”) and negative emotions (i.e. “nervous/anxious,” “worried,” and “stressed”). Therefore, it was rational to infer that the emojis classified in this cluster corresponded appropriately to the emotional state of the core affect in general; however, some emojis require caution because their interpretations can vary.

In the “neutral sentiment with a positive bias” cluster, the following 12 emojis were classified: , , , , , , , , , , , and . Its characteristics comprised a moderate to high valence and a low arousal level (valence: 5.49 ± 0.38, arousal: 5.19 ± 0.29). The human emotional states that were considered to correspond with these levels were “calm,” “relaxed,” and “serene.” The “Relieved face ” emoji was classified in this cluster. However, none of the emojis grouped into this cluster in this study were categorized into the “neutral/dispersed sentiment” cluster in previous research^5^. The “Smiling face with sunglasses ,” “Relieved face ,” and “Face with tongue ” emojis were grouped into the “positive sentiment” cluster; additionally, the previous study’s participants interpreted them using the words “be/act cool,” “happy,” “naughty/playful,” “exited,” and “content/satisfied”^5^. Hence, it can be considered that this cluster’s emojis represented a slightly greater positive emotional state than the valence and arousal levels.

In the “moderately positive sentiment” cluster, the following nine emojis were classified: , , , , , , , , and . Its characteristics consisted of high valence and moderate arousal levels (valence: 6.57 ± 0.62, arousal: 5.98 ± 0.29). The human emotional states that were considered to align with these levels were “contented” and “happy.” Interestingly, all emojis in this cluster had a raised corner of the mouth or smiling eyes (i.e., they were smiling). None of them were classified into the more negative clusters (i.e., “neutral/dispersed sentiment” and “negative sentiment”) in previous studies. Therefore, this cluster’s emojis corresponded well with the emotional states in the core affect.

In the “strong positive sentiment” cluster, the following 12 emojis were categorized: , , , , , , , , , , , . The human emotional states that were considered to correspond to these levels were “elated” and “excited.” All emojis in this cluster had a raised corner of the mouth or smiling eyes (i.e., they were smiling), similar to those classified into the “moderately positive sentiment” cluster. Additionally, many of them contained extra accessories with richer expressions, such as heart (star)-shaped eyes, pink cheeks, or throwing hearts. The “Smiling face with smiling eyes ” emoji was interpreted using the words “happy,” “feeling good,” and “excited” in the previous study^5^. Further, similar to the “moderately positive sentiment” cluster, none of the emojis classified into this one in the present study were grouped into the more negative clusters (i.e., “neutral/dispersed sentiment” and “negative sentiment”) in previous research. Therefore, it was acceptable to deduce that the emojis classified into this cluster corresponded extremely well to the emotional state of the core affect.

The emojis used in this experiment and a previous study^5^ were distributed in a U-shape on the two axes of valence and arousal. With respect to the valence axis, the differences of emojis could be seen in the eyes and mouth shapes. For example, the “Grinning face with smiling eyes ” and the “Angry face ,” which were classified into the “strong positive sentiment” cluster and the “strong negative sentiment” cluster, respectively, have totally different eyes and mouth shapes. These emojis indicate different valence levels (7.70 (1.54) for the former and 2.97 (2.13) for the latter) but similar arousal levels (7.02 (1.52) for the former and 6.80 (2.04) for the latter). On the other hand, the differences of emojis based on the arousal axis were observed to be affected by extra accessories. For example, “Smiling face with heart-eyes ” has extra accessories of heart-shaped eyes compared with “Grinning face with smiling eyes .” Both emojis were classified into the “strong positive sentiment” cluster and indicated similar valence levels (7.78 (1.50) for the former and 7.70 (1.54) for the latter) but had different arousal levels (7.95 (1.39) for the former and 7.02 (1.52) for the latter). These results suggest that people may recognize different valence levels based on the shape of facial parts, and different arousal levels due to the presence of extra accessories. As explained earlier, previous studies reported that the recognition of human expressions is largely based on the eyes, muscular contraction, and other movements^7, 8^. As the emojis used in this study are static anthropomorphic images and cannot represent muscular contractions and other such movements, people may use extra accessories to support their recognition of arousal levels.

It has been reported that the human emotional states (the core affect) were circularly aligned on the valence and arousal axes^4^. Therefore, the emojis, at least those used in the present and previous related studies, may not be able to capture the human emotional states with neutral valence and high arousal levels (i.e., “tense” and “alert”). Jaeger et al.^5^ noted that “open-mouthed facial emoticons (e.g., , ),” which indicate expressions of surprise, may suggest a higher level of arousal. In fact, the arousal levels for these emojis in the present study were somewhat higher than the mean of cluster 4 in which these emojis were included (5.52 and 5.24 respectively, while the mean for cluster 4 was 5.19). Further, as discussed above, adding extra accessory would support the recognition of arousal levels. However, the current emojis could not reach sufficient levels of arousal to capture the “tense” and “alert” emotional states. As the present and prior studies used major facial emojis, it may be difficult to indicate these emotional states with the current set of emojis. Thus, we encourage emoji designers to produce new emojis that can express these human emotional states.

Based on our findings, the fact that emojis can display human emotional states in considerably greater detail than reported previously will accelerate their use in research fields such as consumer studies that can benefit from evaluating these states. This is because the traditional text-based methods can be taxing for the participants^12^, and emojis are considered an easier way to examine human emotional states. In addition, the latter may have the advantage of being less impacted by the participants’ native language than the former. However, this study, as well as prior research on human emotional states expressed by emojis, have many limitations, which are discussed below. Further research is warranted to deepen our understanding of the relationship between emojis and human emotional states; nevertheless, our findings would further increase emoji use in various research areas where human emotional states need to be assessed.

This study has several limitations that must be acknowledged when interpreting the results. First, all participants in this study were young Japanese adults, and individuals with other demographics were not included (i.e., based on age, sex, and culture). Although the “use” of emojis has been reported to be significantly influenced by demographic characteristics such as age, gender, and culture^2^, it has been indicated that their “interpretation” is not significantly impacted by these characteristics^5,13,14^. Therefore, we believe that the results of this study are consistent with other demographics.

Second, we cannot deny the possibility that slight differences in emoji design may have affected the study findings because we only employed the emojis as displayed on Twitter. Even with the same code, the emoji designs displayed on different devices, such as the PC, Mac, Android, and iPhone, vary slightly. Since studies using emojis are conducted with various types of devices, it may be necessary to understand how minor discrepancies in emoji designs displayed on different types of devices affect the interpretation of human emotional states.

Finally, we associated emojis with the human emotional states indirectly based on the valence and arousal axes and the theory of core affect^4^, as the primary purpose of this study was to understand how various facial emojis are classified on these axes. Further research is warranted to directly relate human emotional states with emojis. However, we believe that the results of this study are sufficiently reliable because the interpretation of emojis in each cluster was consistent with that reported by a previous study, which directly linked human emotional states with emojis using open-ended questions.

This study attempted to understand how various facial emojis are categorized on the valence and arousal axes, assess the relationship between these emojis and associated human emotional states, and understand how people recognize arousal and valence levels from emojis. Our results provided evidence that the emojis could be grouped into the following six clusters on the two axes of valence and arousal: (1) a strong negative sentiment, (2) a moderately negative sentiment, (3) a neutral sentiment with a negative bias, (4) a neutral sentiment with a positive bias, (5) a moderately positive sentiment, and (6) a strong positive sentiment. Further, we corresponded each of these clusters with the emotional states described in the core affect theory. Thus, we concluded that emojis display human emotional states in considerably greater detail than previously reported. This finding would accelerate their use in research fields that need to evaluate human emotional states.

## Methods

### Participants

Overall, 1082 individuals aged between 20 and 39 years (*M* age = 30.53, *SD* = 5.30; 532 males and 550 females) living in the capital region of Japan participated in an online survey. They were fluent in Japanese (the language in which the survey was implemented). They registered through an online panel maintained by a marketing research firm (https://www.myvoice.co.jp/voice/). In accordance with the ethical approval obtained prior to commencing data collection, the eligible participants were assured that their responses would remain confidential. This study was conducted in accordance with the World Medical Association’s Declaration of Helsinki, and all study protocols were reviewed and confirmed by the local institutional review board (Committee on Ergonomic Experiments of the National Institute of Advanced Industrial Science and Technology). All participants provided informed consent before their engagement in the study.

### Emojis used in this study

The present research employed human facial emojis, similar to a previous study^5^, because they were the most frequently used categories of emoji. We selected 74 emoticons from the 94 facial emojis registered in Twemoji^15^. We excluded 20 emojis (e.g., , ) based on several comments from the members of our institute during the preliminary survey, who shared that it was difficult to explain emotions associated with these emojis. As emoji designs varied slightly across services (i.e., Twitter, Instagram, Facebook, and WhatsApp), the present study used the emoji designs displayed on Twitter. The emojis were saved as an image file and displayed on an appropriately sized screen (2.16 × 2.16 cm) to ensure that the participants could observe each one clearly.

### Questionnaire

The online questionnaire consisted of two parts. The first one examined the participants’ socio-demographic and background characteristics. The second part evaluated the valence and arousal levels of each emoji using a nine-point scale (1: displeasure to 9: pleasure for valence, and 1: weak to 9: strong for arousal), similar to a previous study^5^. To keep the process less tedious for participants, we requested them to rate only 30 of the 74 emojis individually. There were 16 different pre-defined patterns of the order in which the emojis were presented; furthermore, each participant was randomly assigned to one of them. Consequently, each emoji was assessed by a minimum of 420 participants.

To check whether the participants completed the questions properly, two additional dummy questions were included in the questionnaire, which could be answered easily if the instructions were properly read (e.g., “What is the subject of this questionnaire that you are being asked to answer?”). Dummy questions were displayed following every 10 questions (i.e., questions 11 and 21). Data of participants who responded incorrectly to these questions were excluded from the further analyses.

### Data analysis

To understand how various facial emojis are classified on the valence and arousal axes, we conducted a hierarchical cluster analysis and one-way ANOVA. The Bonferonni correction was used for a post-hoc analysis when the main effect was obtained. The results of the ANOVAs were considered statistically significant if the *p* values were less than 0.05, and if the effect size (*η*2) was greater than 0.06. All statistical analyses were performed using IBM SPSS Statistics 26 (SPSS Inc., Chicago, IL, USA) and the R software^16^.

## Data Availability

The datasets generated during and/or analysed during the current study are available from the corresponding author on reasonable request.
