# Author Correction: Classification of 74 facial emoji’s emotional states on the valence-arousal axes

**DOI:** 10.1038/s41598-024-59799-6

**Published:** 2024-04-18

**Authors:** Gaku Kutsuzawa, Hiroyuki Umemura, Koichiro Eto, Yoshiyuki Kobayashi

**Affiliations:** Human Augmentation Research Center, National Institute of Advanced Industrial Science and Technology, Kashiwa, Japan

Correction to: *Scientific Reports* 10.1038/s41598-021-04357-7, published online 27 January 2022

The original version of this Article contained errors in Table 5, where the values under ‘N’, ‘Valence’ and ‘Arousal’ in ‘Cluster 6’ for the emoji ‘Smiling face’ were interchanged with the values of the same columns for the emoji ‘Smiling face with smiling eyes’.

The correct and incorrect values appear below.

Incorrect:

**Table:** 

	Name	Emoji	N	Valence	Arousal
Cluster6	Smiling face		448	7.75 (1.32)	7.03 (1.47)
	Smiling face with smiling eyes		385	7.50 (1.32)	6.77 (1.53)

Correct:

**Table:** 

	Name	Emoji	N	Valence	Arousal
Cluster6	Smiling face		385	7.50 (1.32)	6.77 (1.53)
	Smiling face with smiling eyes		448	7.75 (1.32)	7.03 (1.47)

The original Article has been corrected.

